# Variable dose interplay effects across radiosurgical apparatus in treating multiple brain metastases

**DOI:** 10.1007/s11548-014-1001-4

**Published:** 2014-04-20

**Authors:** Lijun Ma, Alan Nichol, Sabbir Hossain, Brian Wang, Paula Petti, Rosemin Vellani, Chris Higby, Salahuddin Ahmad, Igor Barani, Dennis C. Shrieve, David A. Larson, Arjun Sahgal

**Affiliations:** 1 Department of Radiation Oncology, University of California, San Francisco, 505 Parnassus Avenue, Room L-08, San Francisco, CA 94143 USA; 2 Department of Radiation Oncology, BC Cancer Agency, University of British Columbia, Vancouver, Canada; 3 Department of Radiation Oncology, University of Oklahoma, Oklahoma City, OK USA; 4 Department of Radiation Oncology, University of Louisville, Louisville, KY USA; 5 Washington Fremont Hospital Gamma Knife Center, Fremont, CA USA; 6 Department of Radiation Oncology, University of Utah, Salt Lake City, UT USA; 7 Department of Radiation Oncology, Sunnybrook Odette Cancer Center, University of Toronto, Toronto, Canada

**Keywords:** Stereotactic radiosurgery, Gamma Knife, Intensity modulation, Brain metastases

## Abstract

**Purpose:**

Normal brain tissue doses have been shown to be strongly apparatus dependent for multi-target stereotactic radiosurgery. In this study, we investigated whether inter-target dose interplay effects across contemporary radiosurgical treatment platforms are responsible for such an observation.

**Methods:**

For the study, subsets ($n = 3, 6, 9,$ and 12) of a total of 12 targets were planned at six institutions. Treatment platforms included the (1) Gamma Knife Perfexion (PFX), (2) CyberKnife, (3) Novalis linear accelerator equipped with a 3.0-mm multi-leaf collimator (MLC), and the (4) Varian Truebeam flattening-filter-free (FFF) linear accelerator also equipped with a 2.5 mm MLC. Identical dose–volume constraints for the targets and critical structures were applied for each apparatus. All treatment plans were developed at individual centers, and the results were centrally analyzed.

**Results:**

We found that dose–volume constraints were satisfied by each apparatus with some differences noted in certain structures such as the lens. The peripheral normal brain tissue doses were lowest for the PFX and highest for TrueBeam FFF and CyberKnife treatment plans. Comparing the volumes of normal brain receiving 12 Gy, TrueBeam FFF, Novalis, and CyberKnife were 180–290 % higher than PFX. The mean volume of normal brain-per target receiving 4-Gy increased by approximately 3.0 cc per target for TrueBeam, 2.7 cc per target for CyberKnife, 2.0 cc per target for Novalis, and 0.82 cc per target for PFX. The beam-on time was shortest with the TrueBeam FFF (e.g., 6–9 min at a machine output rate of 1,200 MU/min) and longest for the PFX (e.g., 50–150 mins at a machine output rate of 350 cGy/min).

**Conclusion:**

The volumes of normal brain receiving 4 and 12 Gy were higher, and increased more swiftly per target, for Linac-based SRS platforms than for PFX. Treatment times were shortest with TrueBeam FFF.

## Introduction

Stereotactic radiosurgery (SRS) is well established as the treatment of choice for patients presenting with 1–4 brain metastases [1, 2]. Clinical data are now emerging to support its role for more than four metastases [3]. This shift in practice from whole brain radiotherapy (WBRT) is largely due to rapid technical developments in SRS technology that enables multiple brain lesions to be treated in a single session [4]. In order to investigate the potential for SRS technologies to adequately treat multiple targets in the brain, we previously evaluated the Gamma Knife Perfexion (Elekta, Atlanta, Georgia), CyberKnife (Accuray, Sunnyvale, California), and Novalis (BrainLAB AG, Heinstetten, Germany) treatment platforms via a multi-center exploratory treatment planning study [5]. In that study, we observed that Perfexion (PFX) yielded the lowest normal brain tissue doses and the benefits increased with increasing number of targets.

However, new technologies have since emerged in the treatment of multiple metastases that require evaluation. The major advance lies in linac-based systems that allow for flattening-filter-free (FFF) and intensity-modulated arc beam delivery. The dominant technology is the TrueBeam FFF (Varian Oncology, Palo Alto, California) system, which is capable of modulating the beam intensity, the dose rate, and the gantry rotation simultaneously during a treatment delivery [6]. This system has been reported as a valid technology in the treatment of multiple metastases targets with a single or few isocenters with rapid treatment times, and a recent clinical study demonstrated promising results specific to the treatment of multiple targets in the brain [7]. Hence, the purpose of our study was to determine how FFF intensity-modulated arc beam SRS technology compares dosimetrically in the treatment of multiple brain metastases to the traditional dynamic conformal arc (DCA) delivery namely as used in the Novalis SRS system, the CyberKnife, and the Gamma Knife PFX.

## Methods and materials

**Fig. 1:**
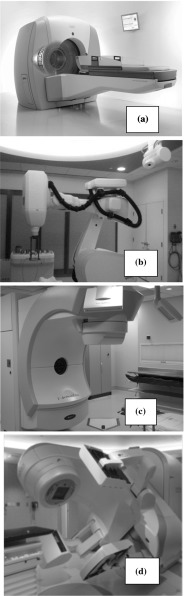
Illustrations of four SRS modalities for the study: **a** Gamma Knife Perfexion **b** Cyberknife **c** Novalis DCA **d** Flattening filter free Truebeam. **a** was courtesy of Elekta (Atlanta, Georgia, USA)

**Fig. 2:**
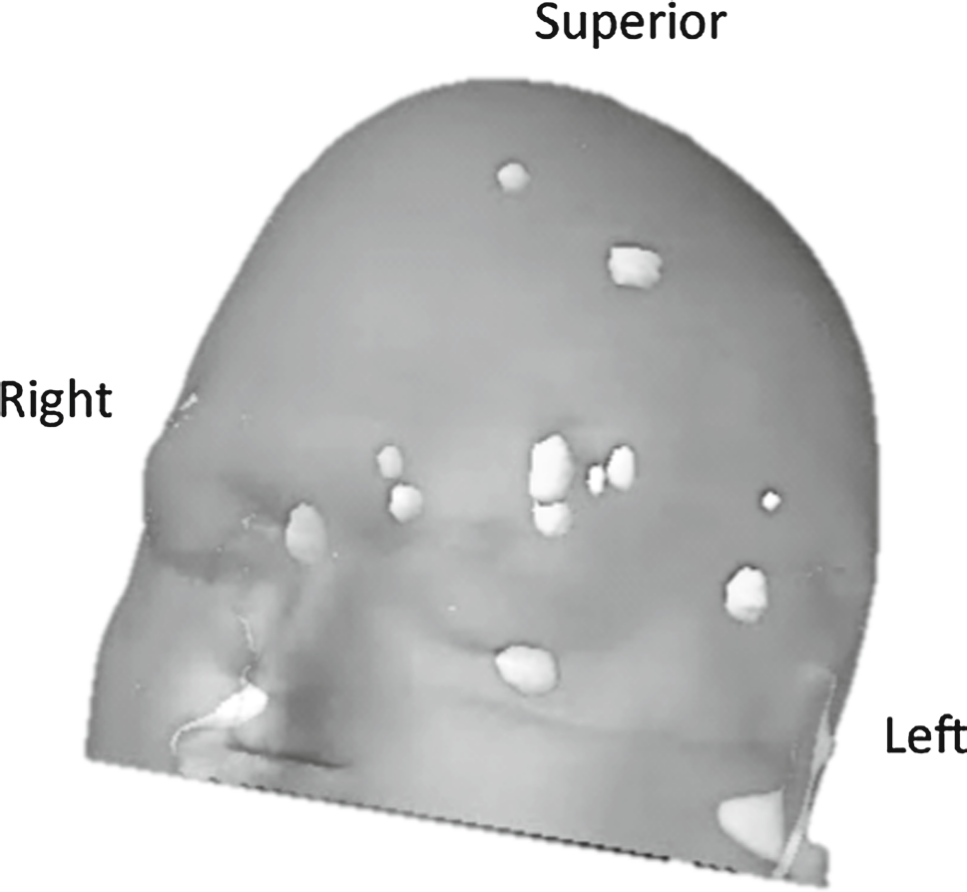
Illustration of the brain metasteses case with 12 lesions within the cranium for the study. All the lesions are 1 cc or less in volume

Following the same multi-institutional study protocol as per our previous publication, a multiple metastases SRS case was selected for independent treatment planning using the Gamma Knife PFX, CyberKnife, Novalis DCA delivery, and the TrueBeam FFF intensity-modulated arc delivery systems. An illustration of these modalities is given Fig. 1. In brief, subsets of target combination with 3, 6, 9, and 12 targets were selected for treatment planning using individual SRS modalities each housed in different institutions, which were chosen due to their expertise with the given technology. The spatial distribution of all the targets is shown in Fig. 2. For a robust comparison of the new TrueBeam FFF with the previously studied modalities, two independent institutions were used for planning with the TrueBeam FFF, each with their own treatment planning strategies.

The dose–volume constraints for the target and the critical structures are given in Table 1, The planning details for Gamma Knife Perfexion, Novalis DCA, and CyberKnife have been described in our previous publication. However, for Cyberknife treatment planning, standard clinical software was used in the current study, as opposed to the research software used in our prior study. The two institutions that performed TrueBeam FFF treatment planning studies used their own independent treatment planning approaches. At one institution, two axial coplanar arcs (one arc rotation followed by another one in reverse direction) were employed for all treatment plans: Arc No 1: 179.9$^\circ$–180.1$^\circ$ with collimator at 45$^\circ$; Arc No 2: 180.1$^\circ$–179.9$^\circ$ with collimator set at 315$^\circ$. At the other institution for the 3-, 6-, and 9-target plans, they used the same approach as above. However, for the 12-target treatment plan, they added one more arc, duplicating Arc No. 2 as Arc No. 3. All plans were created for a high-definition MLC with a 2.5 mm central leaf width.

**Table 1:** Dose–volume histogram-based treatment planning constraints for individual targets and the normal structures for this study

Structure name	Target	PS	PG	BS	ON	OC	Eyes	Lens	Ears
Constraint type	>99 % Volume	Mean dose	Mean dose	Max dose	Max dose	Max dose	Max dose	Max dose	Max dose
Dose level	$\ge$20 Gy	$\le$6 Gy	$\le$8 Gy	$\le$10 Gy	$\le$8 Gy	$\le$8 Gy	$\le$6 Gy	$\le$2 Gy	$\le$4 Gy

*PS* pituitary stalk, *PG* pituitary gland, *BS* brain stem, *ON* optical nerve, *OC* optical chiasm

Each treatment plan was centrally analyzed. The dose–volume parameters for the normal brain as well as other critical structures were extracted. Comparisons were made to investigate (1) peripheral normal brain isodose volume changes for each target combinations and (2) clinical beam-on time for each modality when delivering these treatment plans.

## Results

**Fig. 3:**
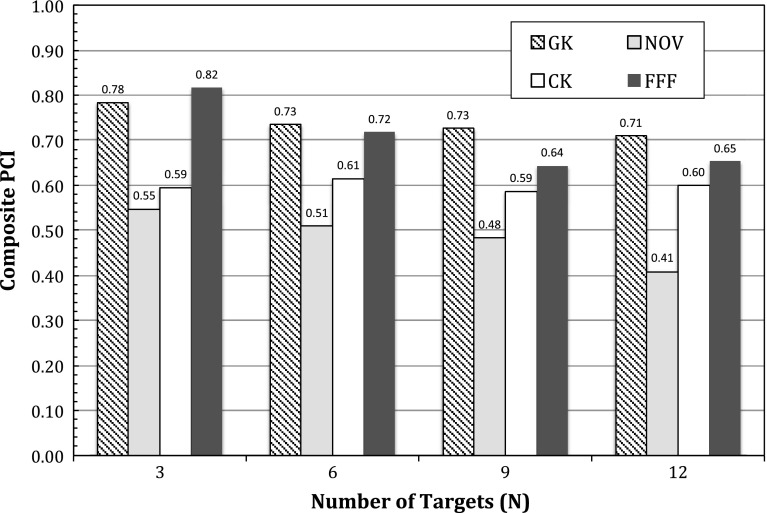
Comparison of Paddick dose conformity index for treatment plans with $N = 3, 6, 9$, and 12 targets respectively. The four SRS modalities are denoted as: *GK* Gamma Knife Perfexion, *CK* CyberKnife, *NOV* Novalis, *FFF* flattening filter free Truebeam

All studied SRS modalities satisfied the normal tissue dose constraints. The lens dose constraint was 2 Gy, and the mean lens doses were 0.31 (range 0.13–0.49) Gy for PFX, 0.37 (range 0.2–0.54) Gy for Novalis, 1.35 (range 0.86–1.84) Gy for CyberKnife, and 1.83 (range 1.68–1.98) Gy for TrueBeam FFF. The higher lens doses observed for the Cyberknife and TrueBeam plans were likely a result of inverse planning optimization to a 2 Gy lens dose constraint. Figure 3 shows the results of Paddick conformity indices (PCI) [9] for the four SRS modalities. Note that Truebeam FFF PCI values were either the highest or the second highest for all the treatment plans. Among isocentric linac-based SRS delivery, Truebeam FFF with intensity-modulated arc beams produced consistently superior PCI values compared with CyberKnife and Novalis DCA delivery (which exhibited the lowest PCI values among the studied SRS treatment modalities). Among all the modalities, the central target dose hot spots such as 120 % of the prescibed dose was highest for PFX because of the standard practice of selecting a low isodose value such as 50 % of the maximum dose when prescribing a treatment.

**Fig. 4:**
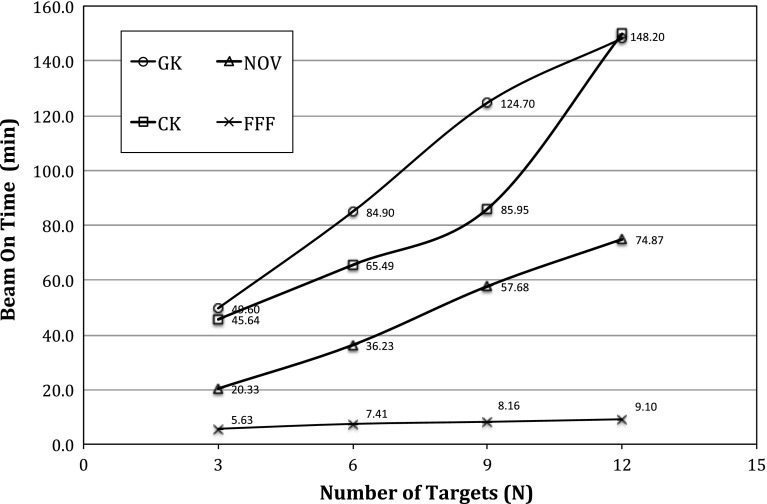
Comparison of the normal beam-on time for treatment plans with $N = 3, 6, 9$, and 12 targets respectively. The four SRS modalities are denoted as: $GK$ Gamma Knife Perfexion, $CK$ CyberKnife, $NOV$ Novalis, $FFF$ flattening filter free Truebeam

The treatment beam-on time for each modality is also plotted in Fig. 4. From the results of Fig. 4, Gamma Knife PFX produced the longest beam-on time, while Truebeam FFF produced the shortest beam-on time. Physically, Gamma Knife PFX delivered treatment at the lowest machine output of 350 cGy/min, while the Truebeam FFF delivery ran at the highest machine output of 1,200 MU/min. Note that the machine output for the Gamma Knife PFX also decreases by approximately 1.1 % per month due to the $^{60}\hbox {Co}$ decay, and the reference dose rate for Cyberknife and Novalis was 800 MU/min in Fig. 4. For the 12-target treatment time as shown in Fig. 4, the treatment time for Cyberknife was found to reach the same level as that of Gamma Knife PFX. This is largely due to the a large number of beam angles required for the Cyberknife cover all the targets simultaneously which in effect lower its dose rate to be comparable to that of Gamma Knife PFX in the clinical version of the planning software [5, 8].

**Fig. 5:**
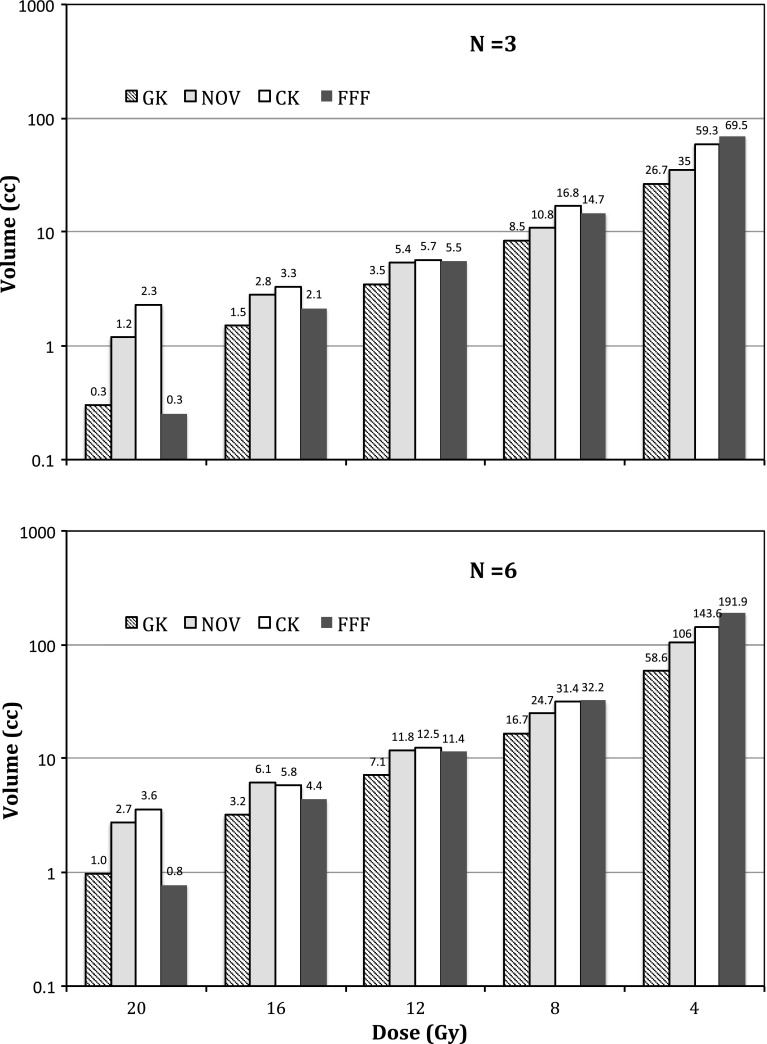
Semi-logarithmic plot of normal brain isodose volumes with $N = 3$ and $N = 6$ of targets for the four SRS modalities ($GK$ Gamma Knife Perfexion, $CK$ CyberKnife, $NOV$ Novalis, $FFF$ flattening filter free Truebeam)

The dependence of varying isodose volumes (i.e., from 20-Gy prescription isodose volume to 4-Gy isodose volume) with increasing number of targets for each SRS modality is plotted in Figs. 5 ($n=3$ vs. $n=6$) and 6 ($n=9$ vs. $n=12$). Note the logarithmic y-axis for all the plots and nonlinear dependence in the isodose volumes with increasing number of targets similar to what was observed in our previous study [5]. Among the four SRS modalities, Gamma Knife PFX yielded the lowest 4-Gy isodose volumes while Truebeam FFF yielded the highest 4-Gy isodose volumes for $n=3, 6, 9$ treatment plans, and CyberKnife yielded the highest 4-Gy isodose volume for $n=12$ treatment plans. The difference in the 4-Gy isodose volumes between the average of these two modalities, and Gamma Knife PFX is in the range of 200–300 %. Although the differences among the four modalities at higher isodose volumes such as the 12-Gy isodose volumes were narrower, Gamma Knife PFX still resulted in the lowest 12-Gy isodose volumes among all the modalities.

**Fig. 6:**
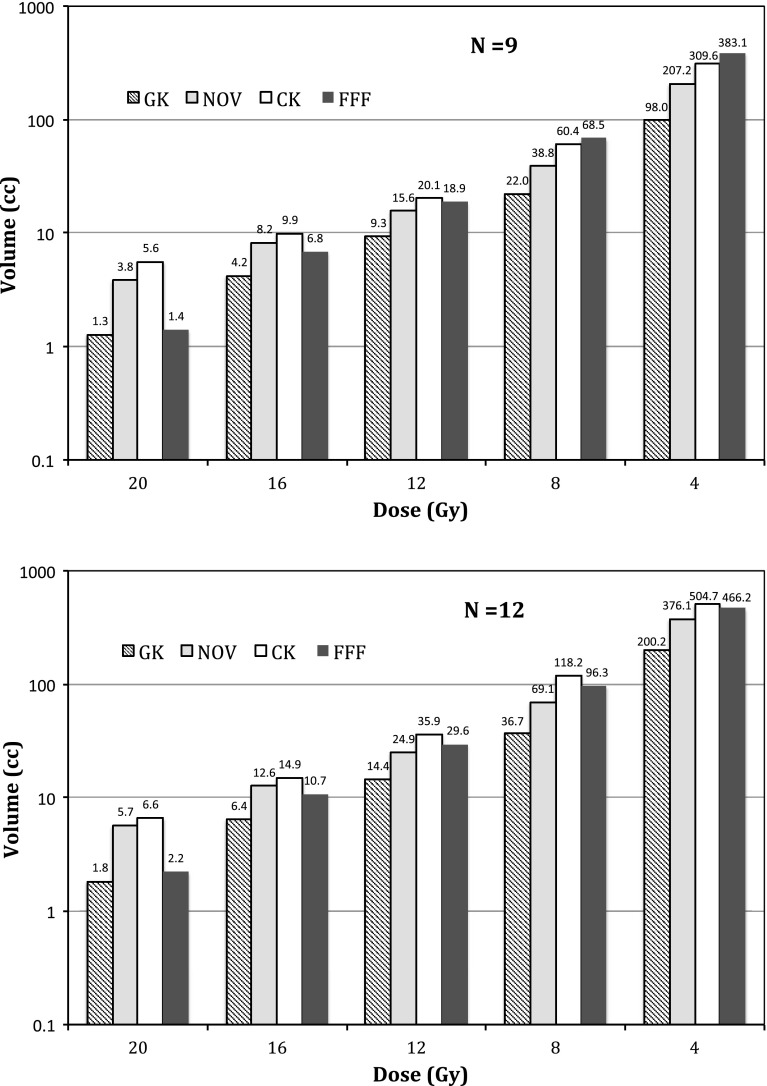
Plot of normal brain isodose volumes with a relatively high number ($N = 9$ and $N = 12$) of targets for the four SRS modalities ($GK$ Gamma Knife Perfexion, $CK$ CyberKnife, $NOV$ Novalis, $FFF$ flattening filter free Truebeam)

**Fig. 7:**
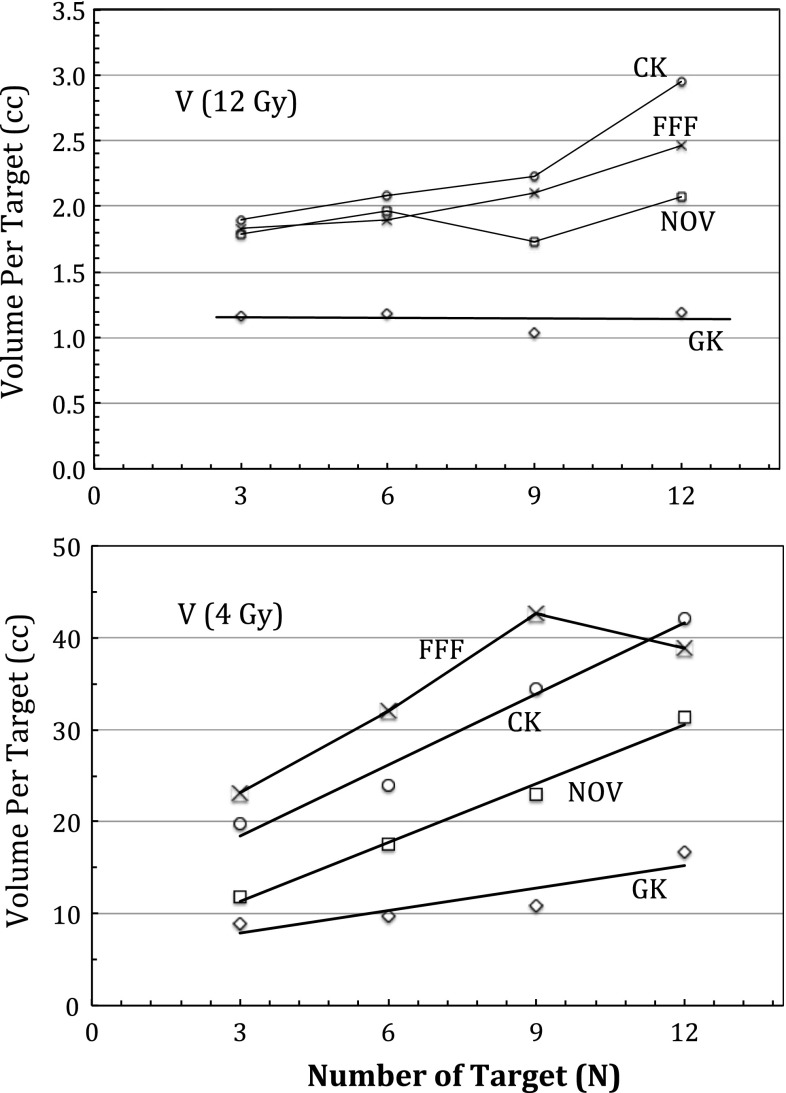
Linear plot of average normal brain isodose volumes per target versus increasing number of targets ($N$) for the four SRS modalities ($GK$ Gamma Knife Perfexion, $CK$ CyberKnife, $NOV$ Novalis, $FFF$ flattening filter free Truebeam). Note the significantly lower slope gradient and y-intercept values for the GK as compared with other linac-based modalities

Finally, dose interplay effects, defined as the additional dose delivered to the normal brain surrounding each target as a result of irradiating the other targets, were measured in this study by examining the average peripheral isodose volumes (such as the dose enclosed by the isodose levels at the 20–80 % of the prescribed dose) per target versus increasing number of targets. Note that such a dose interplay effect was contributed by the scattering as well as the direct-hit photon beams. For example, when planning a three-target SRS case, irradiation of the first target from a large number of beams (e.g., 100–200) inevitably produced a variable dose background to the second and to the third target. However, when accounting for such a dose background, dose delivered to the second or to the third target will in return affect the dose to the first target in a reciprocal manner. As the number of the targets increases, such a dose interplay effect was found to vary among different SRS platforms. The results are illustrated in Fig. 7. Both the 12-Gy and the 4-Gy isodose volumes were plotted with increasing number of targets in Fig. 7. A rapid linear increase in the 4-Gy isodose volume per target, indicating greater interplay effects, was observed for linac-based modalities as compared to the Gamma Knife PFX. The dip for the Truebeam FFF curve at $n=12$ was from averaging the results from the two different treatment planning strategies (e.g., averaging the results between the 2-arc technique and the 3-arc technique), in contrast to those of $n=$ 3-, 6-, 9-target treatment plans where all FFF treatment plans were created with a 2-arc technique. Significantly higher increase in the 12-Gy volume per target was also noted for the linac-based modalities as compared to the PFX, where no dependence in the 12-Gy volume per target with increasing number of targets was observed. This suggests that interplay effects significantly increase the 12-Gy peripheral isodose volume for the linac-based modalities, but less so for the Gamma Knife PFX.

## Discussion

In this study, we report significantly lower normal brain tissue exposure when treating multiple brain metastases with the Gamma Knife Perfexion, as compared to contempory linac-based SRS modalities including the single-isocenter TrueBeam FFF intensity-modulated arc beam-based SRS system. Furthermore, we found that dose interplay effects tracked the trend for these modalities and this is the first report quantifying dose interplay effects for multi-target intracranial SRS treatments.

### Comparison among modalities for multi-target SRS

Based on the dose interplay effect analysis, we observe that multi-isocenter SRS delivery, such as that implemented by the Gamma Knife PFX and Novalis DCA, tends to produce better normal brain tissue sparing as compared to single-isocentric SRS (TrueBeam FFF) or non-isocentric SRS (CyberKnife). In addition, the dose interplay effects were greater at the lower doses, such as the 4-Gy isodose volume, as compared to the higher doses, such as the 12-Gy isodose volume. This is expected as the lower-level isodose volumes tend to be more affected by the scattered radiation plus entrance and exit dose contributions, as compared to the higher-level isodose volumes where primary radiation contributions tend to dominate.

With respect to the beam-on times plotted in Fig. 4, these should be carefully interpreted as their values may grossly underestimate the overall effort involved in the actual treatment. For example, little time gap can be expected between the beam-on time and treatment delivery for Gamma Knife PFX due to the rigid frame alignment and the known output rate from the $^{60}\hbox {Co}$ decay. In comparison, daily output and potential isocenter variability in linear accelerator-based SRS mandate stringent patient setup and machine quality assurance efforts to realize this complex treatment (e.g., machine output verification plus isocenter fidelity checks). In addition, patient alignment with online or near-real time kV imaging must be added to the beam-on times reported in Fig. 4.

### Significance of current work

Unlike our previous exploratory study, where no clinical reports exist for using Novalis DCA or Cyberknife SRS toward multi-target SRS of $n>3$ brain metastases, this work for the first time compared the clinically reported FFF-based SRS technique against the standard Gamma Knife PFX for multi-target SRS in a controlled multi-center setting. The inclusion of Novalis DCA and Cyberknife data was for the purpose of completion and consistency as well as for composite analysis of the newly proposed dose interplay effects across all state-of-the-art SRS platforms. From the dose interplay analysis, it is evident that minimizing inter-target dose cross-talk is the key in decreasing the dose to the normal brain dose for clinical implementation of a new multi-target SRS technology.

Limitations of the current study include that all of the planning was performed on a common set of image studies with limited number of target combinations and distributions. Note that the 12-studied targets were 1.0 cc or less in volume and mostly near spherical. The results of the study may not be applicable for single large or irregularly shaped metastases as shown in another study [10].

It is worth noting that, as in our previous study [5], we have confirmed a general trend in the variations of the normal brain dose among the SRS modalities. We acknowledge that special modification of the linac-based treatment planning techniques, such as expanding solid angles of beam access and/or more beams/arcs, may produce different values than those shown in Figs. 5 and 6. However, this study used expert planners familiar with their own SRS technology, so we surmise that extra planning effort would produce relatively small improvements compared with the large differences observed due to the SRS technologies themselves.

### Clinical relevance and future studies

Theoretically, the greater the radiation exposure of normal brain, the greater the potential for neurocognitive adverse effects. Although it has been confirmed in randomized trials that WBRT result in diminished verbal memory scores and quality of life scores for patients [11–13], the clinical impact of exposing partial volumes of the normal brain to low doses of radiation in a single fraction is unknown. Interestingly, a recent study on hippocampal sparing WBRT suggests less potential for neurocognitive decline. Gondi et al. [14] reported that a dose of $7.3~\hbox {Gy}_{2}$ to 40 % of both hippocampi represents a threshold for cognitive impairment. The biologically equivalent single-fraction dose is 2.9 Gy. Therefore, SRS alone for patients with multiple metastases is likely to yield better neurocognitive outcomes as it is the most efficient in sparing the hippocampi by avoiding WBRT altogether. We also hypothesize that the best sparing of low-dose radiation exposure to the hippocapi will likely be optimal for Gamma Knife PFX given that the maximal benefits occur at the low doses and not at the higher doses.

The other major clinical issue with respect to peripheral isodose exposure is the risk of radionecrosis. A clinically validated relationship has been reported demonstrating that the greater the 12 Gy volume, the greater the risk of radionecrosis [15]. Therefore, it is reasonable to expect that the ability of the Gamma Knife PFX to minimize 12-Gy volume in normal brain may yield lower rates of radionecrosis. This expectation needs clinical validation in the context of SRS treatments for multiple brain metastases. On-going clinical trials such as the randomized study for multiple metastases led by the North American Gamma Knife Consortium and the MD Anderson Cancer Centre Phase 3 randomized trial comparing WBRT to SRS alone for 1–10 metastases may answer these questions.

## Conclusion

For the multi-target SRS, Gamma Knife PFX spares the normal brain tissue best, however, at the expense of longest beam-on time. Linac-based SRS delivery systems yield greater dose exposure within the normal brain tissues. Normal brain exposure per target rises more swiftly with linac-based technologies than Gamma Knife PFX.

## References

[1] Tsao MN, Lloyd N, Wong RK, Chow E, Rakovitch E, Laperriere N, Xu W, Sahgal A (2012) Whole brain radiotherapy for the treatment of newly diagnosed multiple brain metastases. Cochrane Database Syst Rev 4:CD003869. doi:10.1002/14651858.CD003869.pub3 10.1002/14651858.CD003869.pub3 PMC6457607 22513917

[2] Tsao M, Xu W, Sahgal A (2012). A meta-analysis evaluating stereotactic radiosurgery, whole-brain radiotherapy, or both for patients presenting with a limited number of brain metastases. Cancer.

[3] Yamamoto M, Kawabe T, Sato Y, Higuchi Y, Nariai T, Barfod BE, Kasuya H, Urakawa Y (2013). A case-matched study of stereotactic radiosurgery for patients with multiple brain metastases: comparing treatment results for 1–4 $$\text{ vs } \ge 5$$ tumors: clinical article. J Neurosurg.

[4] Grandhi R, Kondziolka D, Panczykowski D, Monaco EA 3rd, Kano H, Niranjan A, Flickinger JC, Lunsford LD (2012) Stereotactic radiosurgery using the Leksell Gamma Knife Perfexion unit in the management of patients with 10 or more brain metastases. J Neurosurg 117(2):237–245. doi:10.3171/2012.4.JNS11870 10.3171/2012.4.JNS11870 22631694

[5] Ma L, Petti P, Wang B, Descovich M, Chuang C, Barani IJ, Kunwar S, Shrieve DC, Sahgal A, Larson DA (2011) Apparatus dependence of normal brain tissue dose in stereotactic radiosurgery for multiple brain metastases. J Neurosurg 114(6):1580–1584. doi:10.3171/2011.1.JNS101056 10.3171/2011.1.JNS101056 21375377

[6] Ling CC, Zhang P, Archambault Y, Bocanek J, Tang G, Losasso T (2008) Commissioning and quality assurance of RapidArc radiotherapy delivery system. Int J Radiat Oncol Biol Phys 72(2):575–581. doi:10.1016/j.ijrobp.2008.05.060 10.1016/j.ijrobp.2008.05.060 18793960

[7] Clark GM, Popple RA, Young PE, Fiveash JB (2010). Feasibility of single-isocenter volumetric modulated arc radiosurgery for treatment of multiple brain metastases. Int J Radiat Oncol Biol Phys.

[8] Ma L, Sahgal A, Hwang A, Hu W, Descovich M, Chuang C, Barani I, Sneed PK, McDermott M, Larson DA (2011). A two-step optimization method for improving multiple brain lesion treatments with robotic radiosurgery. Technol Cancer Res Treat.

[9] Paddick I, Lippitz B (2006). A simple dose gradient measurement tool to complement the conformity index. J Neurosurg.

[10] Ma L, Sahgal A, Descovich M, Chuang C, Huang K, Shrieve DC, Larson D (2010). Equivalence in dose fall-off for isocentric and non-isocentric intracranial treatment modalities and its impact on dose fractionation schemes. Int J Radiat Oncol Biol Phys.

[11] Chang EL, Wefel JS, Hess KR, Allen PK, Lang FF, Kornguth DG, Arbuckle RB, Swint JM, Shiu AS, Maor MH, Meyers CA (2009). Neurocognition in patients with brain metastases treated with radiosurgery or radiosurgery plus whole-brain irradiation: a randomised controlled trial. Lancet Oncol.

[12] Sun A, Bae K, Gore EM, Movsas B, Wong SJ, Meyers CA, Bonner JA, Schild SE, Gaspar LE, Bogart JA, Werner-Wasik M, Choy H (2011). Phase III trial of prophylactic cranial irradiation compared with observation in patients with locally advanced non-small-cell lung cancer: neurocognitive and quality-of-life analysis. J Clin Oncol.

[13] Sperduto PW, Wang M, Robins HI, Schell MC, Werner-Wasik M, Komaki R, Souhami L, Buyyounouski MK, Khuntia D, Demas W, Shah SA, Nedzi LA, Perry G, Suh JH, Mehta MP (2013). A phase 3 trial of whole brain radiation therapy and stereotactic radiosurgery alone versus WBRT and SRS with temozolomide or erlotinib for non-small cell lung cancer and 1 to 3 brain metastases: Radiation Therapy Oncology Group 0320. Int J Radiat Oncol Biol Phys.

[14] Gondi V, Hermann BP, Mehta MP, Tome WA (2013). Hippocampal dosimetry predicts neurocognitive function impairment after fractionated stereotactic radiotherapy for benign or low-grade adult brain tumors. Int J Radiat Oncol Biol Phys.

[15] Lawrence YR, Li XA, el Naqa I, Hahn CA, Marks LB, Merchant TE, Dicker AP (2010). Radiation dose-volume effects in the brain. Int J Radiat Oncol Biol Phys.

